# Risk factors of LEEP margin positivity and optimal length of cervical conization in cervical intraepithelial neoplasia

**DOI:** 10.3389/fonc.2023.1209811

**Published:** 2023-06-22

**Authors:** Qing Cong, Yi Yu, Yu Xie, Yanyun Li, Long Sui

**Affiliations:** Cervical Diseases Diagnosis and Treatment Center, Obstetrics and Gynecology Hospital of Fudan University, Shanghai, China

**Keywords:** LEEP, conization margins, length, transformation zone, cervical intraepithelial neoplasia

## Abstract

**Background:**

The conization length for cervical precancerous lesions is essential for treatment but is left undetermined. This study aims to explore the reasonable and optimal conization length in patients with different types of cervical transformation zones (TZs) to reach the treatment outcome of margin negative in the surgery.

**Methods:**

From July 2016 to September 2019, a multi-center prospective case–control study with or suspicion of cervical precancer was enrolled from five medical centers in Shanghai, China. The clinical characteristics, cytology, human papillomavirus (HPV), histopathology, and details of cervical conization were recorded.

**Results:**

A total of 618 women were enrolled in this study; 6.8% (42/618) had positive internal (endocervical and stromal) margins and 6.8% (42/618) had positive external (ectocervical) margins of loop electrosurgical excision procedure (LEEP) specimen. Comparing the positive internal margin group with the negative group, age (p = 0.006) and cytology (p = 0.021) were significantly different. Multivariate logistic regression analysis showed that the risk factors for positive internal margin were cytology ≥ high-grade squamous intraepithelial lesion (HSIL) (odds ratio (OR) 3.82, p = 0.002) and age (OR 1.11, p < 0.001). The positive internal margin rate was 2.7%, 5.1%, and 6.9% in TZ1, TZ2, and TZ3, respectively, while the positive external margin was 6.7%, 3.4%, and 1.4%, respectively. In the TZ3 group, the HSIL positive internal margin of the 15–16-mm group (10.0%, 19/191) was significantly greater than in TZ1 (2.7%, 4/150) (p = 0.010) and TZ2 (5.0%, 9/179) (p = 0.092); when excision length increases to 17–25 mm, the positive internal margin rate dramatically decreased to 1.0% (1/98).

**Conclusion:**

A cervical excision length of 10–15 mm is reasonable for TZ1 and TZ2 patients, while 17–25 mm is optimal for TZ3 excision with more negative internal margins.

## Introduction

Cervical cancer has high morbidity and mortality across the world, accounting for an estimated 604,000 new cancer cases and 342,000 deaths worldwide in 2018 (1, 2). For cervical precancerous lesion treatment, excision procedures comprise three methods, i.e., loop electrosurgical excision procedure [LEEP; also called large loop excision of the transformation zone (LLETZ)], laser, and cold knife (3, 4).

According to the 2011 International Federation of Cervical Pathology and Colposcopy (IFCPC) terminology, the cervical transformation zone (TZ) is categorized into three types, with the squamous–columnar junction (SCJ) also separated into three types, and it is proposed that the cervical conization length of each transformation zone is different (5). In the 2011 IFCPC colposcopy terminology, TZ3 patients’ conization requires longer and larger excision of cervical tissue than type 1 or type 2 with a significant amount of endocervical epithelium. However, the precise conization length of different TZs was not defined and thus needs further investigation. The 2017 American Society for Colposcopy and Cervical Pathology (ASCCP) terminology categorized SCJ into two types (namely, fully visible SCJ and incompletely visible SCJ) and two types of treatment principles for cervical lesions (6, 7). The British National Health Service (NHS) cervical screening program gives more detailed information for three types of TZs with a wide range of conization lengths, i.e., 7–10, 10–15, and 15–25 mm for TZ1, TZ2, and TZ3, respectively (8).

**Figure 1:**
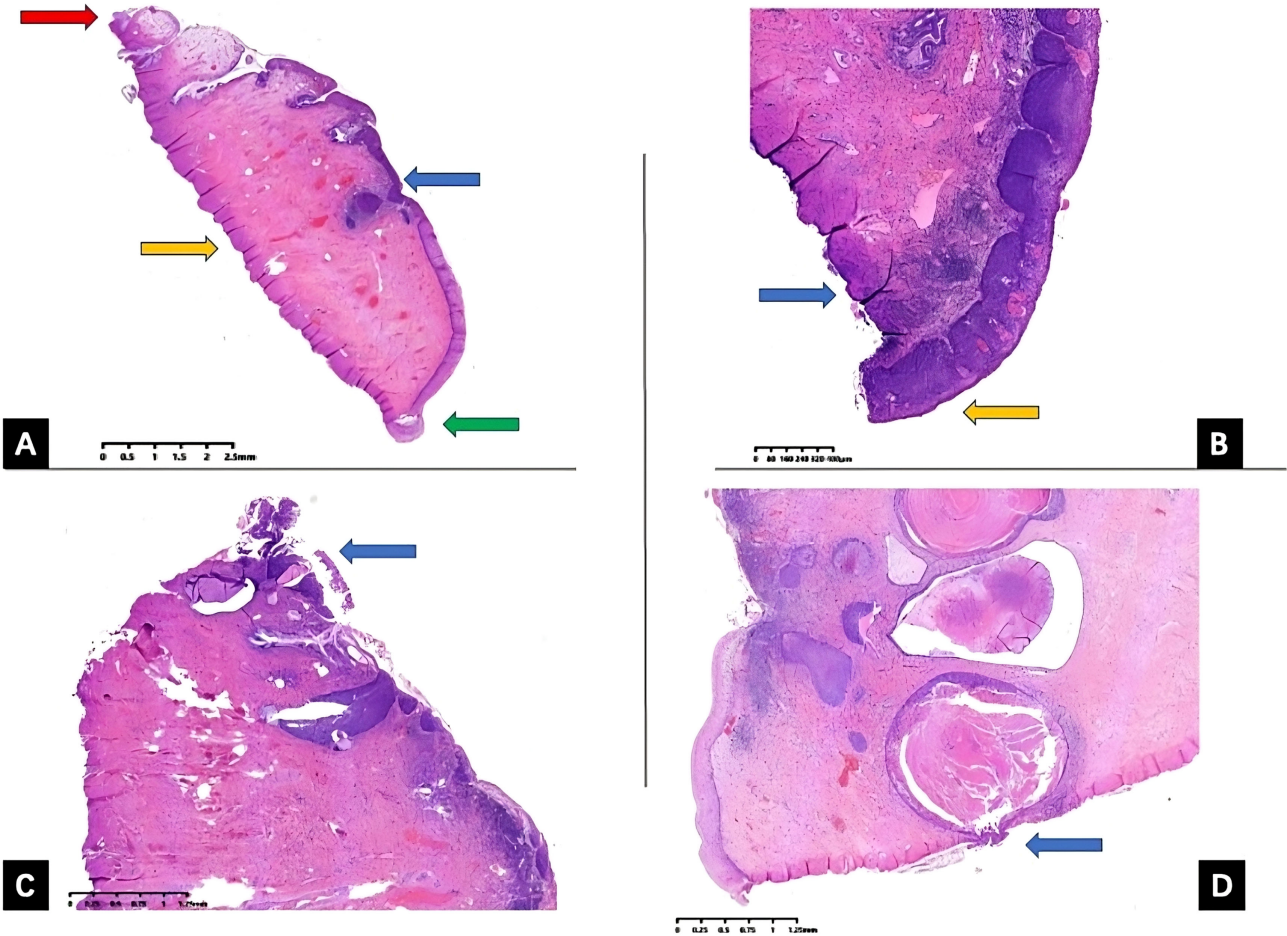
Uterine cervix LEEP specimen histopathology slides. Uterine cervix LEEP conization specimen hematoxylin and eosin (H&E) staining histopathology slides with different margin status. **(A)** All margins are negative, external (ectocervical) margin is demonstrated by green arrow, red arrow pointing at internal (endocervical) margin, orange arrow pointing at the stromal margin, and blue arrow pointing at the HSIL lesions. **(B)** External (ectocervical) margin positive, orange arrow pointing at external margin, blue arrow pointing at the stromal margin **(C)** internal (endocervical) margin positive, blue arrow pointing at the endocervical margin **(D)** internal (stromal) margin positive, and blue arrow pointing at the stromal margin. LEEP, loop electrosurgical excision procedure; HSIL, high-grade squamous intraepithelial lesion.

However, currently, the LEEP conization length has not been standardized (9–11). According to a previous retrospective study carried out in our center in 2019, comparing the different margin status of conization specimens, the persistence rate of endocervical and stromal margin status has no significant statistical difference for internal margins, while internal margin and other margins (ectocervical margins status and negative margin) differ significantly (12). Therefore, to clarify, we group the endocervical and stromal margins as internal margins while the ectocervical margin as external margins, which is also demonstrated in **Figure 1**.

In this study, we investigated cervical conization lengths and margin status in LEEP patients to explore a reasonable excision length for different types of TZs and to help achieve better outcomes of treatment simultaneously.

## Materials and methods

From July 2016 to September 2019, we prospectively enrolled patients with or suspicious of cervical precancer, including 1) cytology high-grade squamous intraepithelial lesion (HSIL)/ASC-H with positive human papillomavirus (HPV) results; 2) colposcopy-directed punch biopsy histological HSIL, regardless of the results of cytology and colposcopy; 3) cytology atypical glandular cells (AGCs) with positive HPV results; 4) colposcopy impression of HSIL with biopsy histopathology low-grade squamous intraepithelial lesion (LSIL). Exclusion criteria: 1) biopsy HSIL with stromal invasion, with invasion depth not determined; 2) cytology HSIL with biopsy confirmed invasive cancer. Cases were included in strict accordance with the inclusion criteria and exclusion criteria.

A total of 618 cases were included from five medical centers, i.e., Obstetrics and Gynecology Hospital of Fudan University, Renji Hospital Affiliated to Shanghai Jiaotong University School of Medicine, Shanghai First Maternal and Child Health Hospital Affiliated to Tongji University, Cancer Hospital Affiliated to Fudan University, and Shanghai Tongji Hospital. According to the LEEP histopathological report, the negative excision margin group includes cervical chronic inflammation, LSIL, HSIL with a negative margin, and adenocarcinoma *in situ* (AIS) with a negative resection margin. The margin with LSIL belonged to a negative margin. Margins equal to or higher than HSIL (HSIL, AIS, and malignancy) belonged to the positive margins. Five types of margin status were analyzed, including positive external, positive internal (endocervical or stromal), positive undetermined, and negative margin.

Stata 15.0 statistical software was used to analyze the data. t-Test and chi-square test analysis were used to analyze the differences in the mean value and proportion, respectively. The logistic regression model was used for univariate and multivariate analyses. p < 0.05 was considered statistically significant.

### Institutional review board statement

This study was conducted in accordance with the Declaration of Helsinki and approved by the ethics committee of the Obstetrics and Gynecology Hospital of Fudan University (IRB Number: 2016-03).

## Results

### Clinical characteristics of 618 women who underwent cervical LEEP

A total of 618 cases of women who underwent cervical LEEP were classified as reported in **Table 1**. The clinical characteristics, including age, parity, mode of delivery, smoking, age of first sexual intercourse, contraceptive methods, cytology, HPV testing, histopathological results, colposcopy examination (type of transformation zones and colposcopy impression), and details of cervical conization (excision length, width, cervical length, the proportion of excision) were all analyzed and compared between two groups (positive internal margins *vs.* negative external margins). Age and cytology were significantly different between the two groups (p = 0.006, p = 0.021).

**Table 1:** Clinical characteristics of 618 women who underwent cervical LEEP.

Clinical characteristics	Total n (%)	Negative external margins (n = 576)	Positive internal margins (n = 42)	p-Value
Age (mean ± SD, years)	38.2 ± 9.1	37.9 ± 9.0	42.0 ± 9.6	0.006*
<30	72	68	4	
30–40	301	282	19	
40–50	187	174	13	
>50	58	52	6	
Age of first sex intercourse	22 ± 2.9	22 ± 2.9	21.2 ± 2.6	0.181
Number of sex partners	1.2 ± 0.8	1.3 ± 0.8	1.2 ± 0.4	0.483
Parity				0.292
0	132	123	7	
1	347	320	24	
≥2	133	122	11	
Delivery mode				0.692
Vaginal delivery	364	336	25	
Cesarean section	248	229	17	
Condom				0.018
Yes	317	303	14	
No	301	273	28	
Smoke				Omitted
Yes	3	3	0	
No	614	572	42	
Cytology				0.021*
NILM	216	203	13	
ASC-US	112	110	2	
LSIL	87	81	6	
ASC-H	71	68	3	
HSIL	114	98	16	
SCC	2	2	0	
AGC	2	2	0	
HPV				0.592
Negative	22	20	2	
Positive	530	497	33	
Transformation Zone				0.140
Type 1	150	144	6	
Type 2	179	166	13	
Type 3	289	266	23	
Colposcopy impression				0.883
Normal	23	22	1	
LSIL	65	59	6	
HSIL	522	487	35	
Suspicion of cancer	1	1	0	
Histopathology of biopsy				omitted
Normal	4	4	0	
LSIL	19	19	0	
HSIL	594	553	41	
Cancer	1	0	1	
Length of cervix (mm)	29.4 ± 4.0	29.4 ± 3.8	29.2 ± 4.2	0.821
Proportion of excision (%)	51.7 ± 9.2	51.7 ± 9.2	51.9 ± 8.9	0.907
Length of excision (mm)				>0.999
<15	105	101	4	
15	388	356	32	
>15	125	119	6	

NILM, negative for intraepithelial lesion or malignancy; ASC-US, atypical squamous cells of undetermined significance; LSIL, low-grade squamous intraepithelial lesion; ASC-H, atypical squamous cells, cannot exclude HSIL; HSIL, high-grade squamous intraepithelial lesion; AGC, atypical glandular cells; LEEP, loop electrosurgical excision procedure. * (P<0.05) indicate a statistically significant difference between the two groups.

### Histopathological results of 618 women who underwent LEEP

According to the histopathological report of LEEP conization of the cervix, 90.0% of women had negative margins, including chronic inflammation 12.3%, LSIL 14.9%, HSIL 62.3%, AIS 0.3%, and HSIL combined with AIS 0.2%; 10.0% of women had positive margins, including HSIL with positive external margin 3.2%, HSIL with positive endocervical margin 4.2%, HSIL with positive stromal margin 1.1%, AIS margin positive 0.3%, AIS combined with HSIL (AIS and HSIL) margin positive 0.3%, squamous cell carcinoma 0.6%, and adenocarcinoma 0.2% (**Table 2**).

**Table 2:** Histopathological results of 618 women who underwent LEEP.

Histopathological results	Number	Percentage
Chronic inflammation	76	12.3%
LSIL	92	14.9%
HSIL	431	69.7%
Negative margin	385	62.3%
Positive ectocervix	20	3.2%
Positive endocervix	26	4.2%
Positive stromal	7	1.1%
AIS	7	1.1%
Negative margin	2	0.3%
Negative margin with HSIL and AIS	1	0.2%
Positive margin	2	0.3%
Positive margin with HSIL and AIS	2	0.3%
Squamous cell carcinoma	4	0.6%
Adenocarcinoma	1	0.2%
Total	618	100%

LSIL, low-grade squamous intraepithelial lesion; HSIL, high-grade squamous intraepithelial lesion; AIS, adenocarcinoma in situ; LEEP, loop electrosurgical excision procedure.

### Risk factors of positive LEEP margins

The patients were categorized into two groups according to the negative and positive cervical LEEP excision margins. The clinical characteristics of the two groups were compared by univariate logistic regression. The results showed the age of first sexual intercourse (p = 0.181), number of sexual partners (p = 0.483), parity (p = 0.292), delivery method (p = 0.692), HPV (p = 0.592), transformation zone (p = 0.140), colposcopy impression (p = 0.883), colposcopy-directed punch biopsy histopathology (p is omitted), cervical length (p = 0.821), conization length (p > 0.999), and the ratio of conization length to cervical length (p = 0.907) were not significantly different between the two groups. However, the difference in cytology (p = 0.021) and age (p = 0.006) in the two groups was statistically significant.

As shown in **Table 3**, multiple variables were then incorporated into the multivariate logistic regression model, including age, age of first sexual intercourse, number of sex partners, parity, vaginal delivery, condom, positive high-risk HPV (hrHPV), transformation zone, cytology ≥ HSIL (ASC-H, HSIL, AGC, AIS, squamous cell carcinoma, and adenocarcinoma), colposcopy impression ≥ HSIL (HSIL, AIS, and suspicious cancer), length of excision, length of the cervix, and proportion of excision. Multivariate logistic regression analysis showed that the risk factors for the positive internal LEEP margin were cytology ≥ HSIL (odds ratio 3.82, 95% confidence interval 1.62–8.97, p = 0.002) and age (odds ratio 1.11, 95% confidence interval 1.05–1.17, p < 0.001).

**Table 3:** Multivariate logistic regression of risk factors of positive LEEP margins.

Candidate variables	Odds ratio	Standard error	95% Confidence interval		p-Value
Cytology ≥ HSIL	3.82	1.66	1.62	8.97	0.002*
Age	1.11	0.03	1.05	1.17	<0.001*
Age of first sexual intercourse	0.86	0.07	0.74	1.01	0.060
Number of sex partners	0.83	0.23	0.49	1.42	0.497
Parity	1.09	0.33	0.60	1.97	0.775
Vaginal delivery	1.61	0.53	0.84	3.09	0.153
Condom	0.91	0.23	0.55	1.51	0.723
Positive hrHPV	0.77	0.68	0.14	4.35	0.770
Transformation zone	1.22	0.39	0.65	2.30	0.533
Colposcopy impression	0.60	0.31	0.22	1.63	0.312
Length of excision	1.19	0.64	0.42	3.42	0.743
Length of cervix	0.90	0.25	0.53	1.55	0.717
Proportion of excision	0.91	0.14	0.68	1.23	0.550

LEEP, loop electrosurgical excision procedure; HSIL, high-grade squamous intraepithelial lesion; hrHPV, high-risk human papillomavirus. * (P<0.05) indicate a statistically significant difference between the two groups.

### LEEP margins according to excision lengths of three transformation zones

In this study, the conization length of the type 1 transformation zone was 13.5 ± 2.3 mm (7–15 mm), the conization length of the type 2 transformation zone was 13.7 ± 2.1 mm (10–15 mm), and the conization length of the type 3 transformation zone was 16.4 ± 2.0 mm (15–25 mm). Type 3 transformation zone patients were subdivided into two groups according to the length of cervical conization as 15 and 16–25 mm for data analysis. The total positive rate of excision margin was 10.7% (16/150), 10.6% (19/179), and 9.3% (27/289). Since positive external margin only has a close correlation with the width of the lesion and no correlation on the residual recurrence extended into the cervical canal, we analyzed the proportion of positive internal margins showing 2.7% (4/150), 5.1% (9/179), and 6.9% (20/289), respectively. Comparing the three groups, the difference was not statistically significant in the positive internal margin rate for TZ1 *vs.* TZ2 (p = 0.274) and TZ1 *vs.* TZ3 (p = 0.063). In TZ3 excision length groups of 15 and 16–25 mm, the positive internal margin positive rate was 9.7% (16/164) and 3.2% (4/125), respectively. When comparing the TZ3 (15 mm) group with the TZ1 group, the difference was statistically significant (p = 0.010). The difference between TZ3 (15 mm) *vs.* TZ2 was not significant (p = 0.092). Neither the difference for TZ3 (16–25 mm) *vs.* TZ1 (p = 0.764) nor TZ3 (16–25 mm) *vs.* TZ2 (p = 0.438) was statistically significant. When excision length was 15, 16, 17, 18, 19, 20, 21, and 25 mm in TZ3, the positive rate of internal resection margin was 9.8% (16/164), 11.1% (3/27), 0% (0/9), 0% (0/47), 0% (0/1), 2.7% (1/37), 0% (0/1), and 0% (0/3), respectively (**Table 4**).

**Table 4:** LEEP margins according to excision lengths of three transformation zones.

LEEP margins	Excision length		
	TZ1 (n = 150)	TZ2 (n = 179)	TZ3 (n = 289)
	7–15 mm	10–15 mm	15–25 mm
Negative	134 (89.30%)	160 (89.4%)	262 (90.66%)
Normal	17 (11.30%)	21 (11.7%)	38 (13.15%)
LSIL	28 (18.70%)	35 (19.6%)	29 (10.03%)
HSIL	88 (58.70%)	103 (57.5%)	194 (67.13%)
AIS*	1 (0.70%)	1 (0.6%)	1 (0.35%)
Positive external: HSIL	10 (6.70%)	6 (3.4%)	4 (1.38%)
Positive endocervical: HSIL	1 (0.70%)	6 (3.4%)	19 (6.57%)
Positive stromal: HSIL	3 (2.00%)	3 (1.7%)	1 (0.35%)
Positive margin: AIS*	1 (0.70%)	2 (1.1%)	1 (0.35%)
Cancer	1 (0.70%)	2 (1.1%)	2 (0.69%)
Total	150 (100.00%)	179 (100.00%)	289 (100.00%)

LSIL, low-grade squamous intraepithelial lesion; HSIL, high-grade squamous intraepithelial lesion; AIS, adenocarcinoma in situ; LEEP, loop electrosurgical excision procedure.

*AIS includes with or without concurrent HSIL.

**Figure 2:**
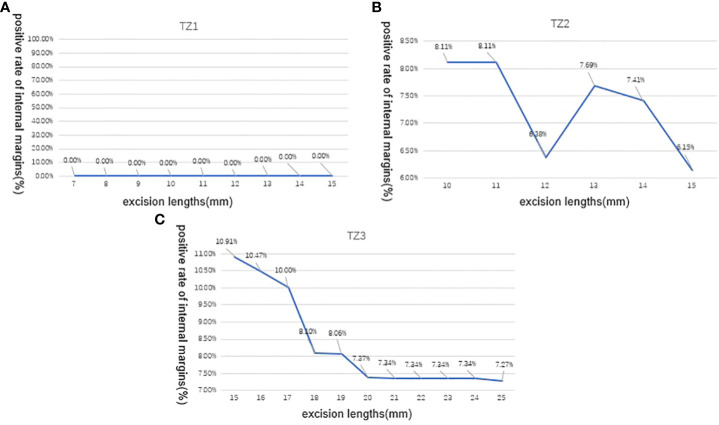
Positive rate of LEEP internal margins according to excision lengths of three transformation zones (TZs). **(A)** In TZ1, the detection rate of positive internal margin was still 0% When the excision length is gradually increased from 7 to 15mm; **(B)** In TZ2, the positive internal margin rate was 8.11%,8.11%, 6.38%, 7.69%, 7.41%, and 6.15% when the excision length is gradually increased from 10 to 15mm, respectively; **(C)** In TZ3, the positive internal margin rate decreased from 10.91% to 7.27% when the excision length is gradually increased from 15 to 25 mm.TZ, transformation zone; LEEP, loop electrosurgical excision procedure.

In **Figure 2**, we evaluated LEEP margins according to the excision lengths of three transformation zones. This figure illustrated the positive rate of internal margins when we gradually added the excision length during conization surgery. In TZ1, when excision length reaches 15 mm, the detection rate of positive internal margin was still 0%; in TZ2, the positive rate was 8.11%, 8.11%, 6.38%, 7.69%, 7.41%, and 6.15% when excision length reaches 10, 11, 12, 13, 14, and 15 mm, respectively. In TZ3, when excision length increased from 15 to 25 mm, the positive internal margin rate decreased from 10.91% to 7.27%, except for one case of HSIL combined with AIS; even if the resection length was 15 mm, the resection margin was positive.

## Discussion

This multi-center prospective study recruited patients who underwent LEEP conization (13–15). After reviewing their histopathological results, the LEEP specimen margin was negative for 90.0% (556/618), and the margin was positive for 10.0% (62/618), 0.81% (5/618) of which were further diagnosed as invasive carcinoma. In this study, most patients with cervical lesions (90.0%) can obtain early diagnosis and precise treatment through a single LEEP conization. Patients with positive margins (9.2%) were triaged to follow-up within 3–6 months after surgery and repeat conization if necessary (16, 17). As a result, most patients can preserve their cervix through cervical conization, without the need for total hysterectomy. Red House Hospital’s single-center retrospective large sample data showed that LEEP specimen histopathology diagnosed 5.97% (759/12,713) as early cervical cancer (18), and the retrospective single-center data from Cancer Hospital affiliated to Fudan University of LEEP specimen histopathology diagnosed 8.60% (112/1,303) as early cervical cancer (19). LEEP specimen histopathology diagnosis rate of cervical cancer (0.81%) was lower than in that of two single centers. The reason might be that this study excluded patients with biopsy results suggesting cervical cancer with an uncertain depth of invasion, and this group of patients was found to have high proportions of invasive cervical cancer diagnosed by conization 61.9%.

Multivariate logistic regression analysis further confirmed that cytology ≥ HSIL and age are high-risk factors for LEEP positive internal margins, which indicates that the severity and duration of the disease might be related to the positive internal margin status. During the 3–6 months’ follow-up after conization, our previously published data and the cancer hospital team’s data showed that age, menopause, positive conization margin, diagnosis of microinvasive carcinoma by conization, abnormal cytology in postoperative follow-up, and positive HPV were high-risk factors for persistent HSIL. In the 3–6 months’ follow-up after conization, the proportion of persistent HSIL in patients with positive margin was 7.6% (46/609) and only 1.9% in patients with negative margin (55/2,964). The risk of persistent HSIL varies in the different positive margins, with positive external margin in 4.7% (13/278), positive undetermined (including stromal) margin in 9.7% (22/227), and positive endocervical margin in 13.2% (23/174). The data show that positive margins do not mean that lesions always persist after surgery, and positive internal margin had significantly higher risks of persistent HSIL when compared with positive external margin. During the 3–6 months’ follow-up after conization, only 7.6% of patients have persistent HSIL after conization on average, which means that up to 92.4% of patients with positive margins for HSIL lesion regressed naturally.

In this study, the LEEP margin of type 1, 2, and 3 transformation zones under different conization lengths was thoroughly investigated. In the condition of excision length of 7–15 mm for TZ1 and 10–15 mm for TZ2, this study showed that both the total positive margin rates and positive rate of external margin of TZ1 were similar to those of TZ2 (10.7% (16/150) *vs.* 10.6% (19/179), and 2.7% (4/150) *vs.* 5.1% (9/179), respectively). Hence, clinicians can simply choose a loop with an excision length of 10–15 mm for fully visible SCJ (TZ1 and TZ2) patients.

In 27 patients with TZ3 with positive margins, most (74.1%) were found when the excision length was 15 mm, and only 26.0% were found in the 16–25-mm group. Both the total positive margin rate and positive internal margin rate of the 15-mm group were significantly higher than those of the 16–25-mm group (12.2% (20/164) *vs.* 5.6% (7/125), 9.7% (16/164) *vs.* 2.7% (4/150)), showing that the excision length of TZ3 should be greater than 15 mm, and the clinician should choose loops with excision length greater than 15 mm. When excision length was 15, 16, 17, 18, 19, 20, 21, and 25 mm in TZ3, the positive rate of internal margin was 9.8% (16/164), 11.1% (3/27), 0% (0/9), 0% (0/47), 0% (0/1), 2.7% (1/37), 0% (0/1), and 0% (0/3), respectively. Hence, 17–20 mm can achieve more negative internal margins and retain a more normal cervix and thus might be optimal for TZ3 excision.

According to the trend line in **Figure 2**, excision of 7–10 mm of the type 1 transformation zone patients is reasonable and safe for HSIL, except for the case of HSIL combined with AIS. If cytology and colposcopy suggest glandular disease, the excision depth needs to be >10 mm. For women who have completed childbirth, the excision length can reach 18–20 mm. The treatment of AIS should follow the latest Society of Gynecologic Oncology Evidence-Based Review and Recommendations of Adenocarcinoma in Situ (20–22).

The limitation of the study is that the sample size of each excision length was not evenly distributed in TZ3, while relatively limited cases were in the 17-mm, 19-mm, and longer than 20-mm groups. In the future, more data from these groups will help to achieve a more accurate conclusion.

In conclusion, the cervical LEEP aims to resect cervical precancerous lesions. However, up to now, the study of conization length is scant. In this study, we explored the reasonable and optimal conization length in patients with different types of TZs. The ideal cervical conization length for patients with TZ1 and TZ2 is 10–15 mm, while 17–25 mm is optimal for TZ3 excision with more negative internal margins.

## Data availability statement

The original contributions presented in the study are included in the article/supplementary material. Further inquiries can be directed to the corresponding author.

## Author contributions

QC and YY had full access to all data and take responsibility for the integrity of the data and the accuracy of the analysis. Study concept and design: LS. Acquisition, analysis, and interpretation of data: QC, YY, YX, and YL. Drafting of the manuscript: QC and YY. Critical revision of the manuscript for important intellectual content: all authors. All authors contributed to the article and approved the submitted version.
